# Transcript-level annotation of Affymetrix probesets improves the interpretation of gene expression data

**DOI:** 10.1186/1471-2105-8-194

**Published:** 2007-06-11

**Authors:** Hui Yu, Feng Wang, Kang Tu, Lu Xie, Yuan-Yuan Li, Yi-Xue Li

**Affiliations:** 1 Shanghai Center for Bioinformation Technology, Shanghai 200235, P. R. China; 2 School of Life Science and Technology, Shanghai Jiaotong University, Shanghai 200240, P. R. China; 3 Key Laboratory of Systems Biology, Shanghai Institutes for Biological Sciences, Chinese Academy of Sciences, Shanghai 200031, P. R. China; 4 Bioinformatics Center, Shanghai Institutes for Biological Sciences, Chinese Academy of Sciences, Shanghai 200031, P. R. China

## Abstract

**Background:**

The wide use of Affymetrix microarray in broadened fields of biological research has made the probeset annotation an important issue. Standard Affymetrix probeset annotation is at gene level, i.e. a probeset is precisely linked to a gene, and probeset intensity is interpreted as gene expression. The increased knowledge that one gene may have multiple transcript variants clearly brings up the necessity of updating this gene-level annotation to a refined transcript-level.

**Results:**

Through performing rigorous alignments of the Affymetrix probe sequences against a comprehensive pool of currently available transcript sequences, and further linking the probesets to the International Protein Index, we generated transcript-level or protein-level annotation tables for two popular Affymetrix expression arrays, Mouse Genome 430A 2.0 Array and Human Genome U133A Array. Application of our new annotations in re-examining existing expression data sets shows increased expression consistency among synonymous probesets and strengthened expression correlation between interacting proteins.

**Conclusion:**

By refining the standard Affymetrix annotation of microarray probesets from the gene level to the transcript level and protein level, one can achieve a more reliable interpretation of their experimental data, which may lead to discovery of more profound regulatory mechanism.

## Background

Microarray technology was invented to rapidly profile the quantities of mRNA transcripts in a particular cellular context [1-3]. Its application has become universal in biomedical researches. Although it is mRNA that is actually detected by microarray experiments, and it is mRNA that has the direct relationship with protein, the methodology and algorithms for data analysis are commonly gene based. As evident in the probe annotation file provided by Affymetrix, gene-level annotation is widely accepted even though it fails to discriminate multiple mRNAs transcribed from the same gene. As a result, the analysis results are usually summarized at the gene level, such as differentially expressed genes [4-6] or gene-sets [7,8]. Even in the recent works that integrate protein-protein interaction data and microarray data [9-11], the analysis unit is reduced to the gene instead of mRNA. This practice could be attributed to the fact that most of the functional knowledge is at gene level instead of transcript level, and the functional differences between mRNA variants transcribed from the same gene are seldom clear. Although the gene-level analysis ignores the difference among mRNAs variants, this strategy is still biologically meaningful considering that the diversity of genes is much higher than that of transcripts encoded by the same gene.

It has been well established that alternative splicing increases mRNA diversity, and over 60% of human genes are involved in this mechanism [12]. In addition, other RNA processing events, such as RNA editing, also account for the increased diversity at the mRNA level [13]. Since these events enable one gene to encode multiple proteins which might be functionally heterogeneous, we feel it necessary to separate transcript-level synonymous probesets from gene-level synonymous ones. The probesets that hybridize to more than one transcript variant of the same gene are referred to as gene-level synonymous probesets; while the ones that hybridize to a single variant are named as transcript-level synonymous probesets. It has been noticed that transcript-level synonymous probesets tend to have similar expression profiles, while gene-level synonymous probesets may have distinct expression profiles [14,15]. In fact, individual reports demonstrated that the expression of transcript variants could be totally different [16,17]. These phenomena indicate that the gene-level strategy of microarray data analysis is imprecise enough that one may overlook the expressional inconsistencies among gene-level synonymous probesets.

As a matter of fact, Affymetrix suffixes their probeset ID according to the probeset's specificity. For example, probesets that recognize unique transcript variants are suffixed with _at, and probesets that recognize multiple alternative transcripts from a single gene are suffixed with _a_at or _s_at, and so on. This suffix system gives a hint on the varied specificities of the probesets, and could be considered as an endeavor trying to do away with customers' worry about the gene-level data analysis strategy. However, the correctness of the suffix system has been in doubt [14,18]. Therefore it is not reliable to perform transcript-level analysis on the basis of this imperfect suffix system. As the standard annotation files and most of the analysis algorithms are gene-oriented, analysts often average out the expression heterogeneity of the same gene when dealing with probeset level data [19].

In this paper, we linked the probesets of two widely used Affymetrix arrays with the International Protein Indexes (IPIs) [20,21] through proper association and rigorous alignment procedures, and demonstrated the statistically significant advantage of interpreting microarray data at the transcript-level or protein-level. Our results can be viewed as a more precise annotation of Affymetrix array's probesets, with which one may achieve a more reliable interpretation of their experimental data. Moreover, the application of this new annotation substantially increased the expression correlation between interacting proteins.

## Results

### Transcript-level or protein-level annotation of Affymetrix probesets

Two Affymetrix arrays, MOE430A_2 and HG-U133A, were chosen for this study, in which 21,097 and 16,213 putative protein-coding probesets for the two arrays were subject to the alignment investigation (Table 1). Candidate probeset-mRNA relationships were compiled based on the probeset-gene mapping information specified in the standard Affymetrix annotation files and gene-mRNA mapping relationships provided by separate mRNA transcript sources. Rigorous blast procedure similar to that described in [22] were performed to filter these candidate probeset-mRNA relationships. Finally, the mRNA targets passing the filtering criteria were linked to protein IDs in the IPI database.

**Table 1:** Summary of probesets, genes and proteins covered by probesets.

	MOE430A_2	HG-U133A
All probesets	22690	22283
Non-control probesets	22626	22215
Probesets associated with protein-coding mRNAs (Investigated probesets)	21097	16213
Probesets passing BLAST (Annotated probesets)	18894 (probeset retaining percentage: 83.5% of the non-control, 89.6% of the investigated)	15288 (probeset retaining percentage: 68.8% of the non-control, 94.3% of the investigated)

Genes covered by non-control probesets	13259	12849
Genes covered by investigated probesets	12853	11286
Genes covered by annotated probesets	12626 (Gene retaining percentage: 95.2% of the non-control) (Gene coverage: 45.1% of all protein-coding genes*)	10976 (Gene retaining percentage: 85.4% of the non-control) (Gene coverage: 43.2% of all protein-coding genes*)

Proteins covered by annotated probesets	21512 (Protein coverage: 42.0% of all proteins*)	22740 (Protein coverage: 40.8% of all proteins*)

*As indicated by the statistics of IPI database (release 3.17), there are totally 51,251 mouse protein entries encoded by 27,992 genes, and 55,782 human proteins encoded by 25,393 genes.

Through the rigorous association and alignment, we obtained precise annotations for 18,894 and 15,288 probesets in Affymetrix arrays MOE430A_2 and HG-U133A respectively (see Additional file 1). These annotations discriminate alternative mRNA variants transcribed from a same gene, thus are at transcript level as opposed to the standard gene-level annotation files provided by Affymetrix. It is worth noting that since the transcript data we used were quite redundant, a conceptual transcript variant may be represented by multiple redundant transcript accessions in the transcript database. In our transcript-level annotation file, each conceptual transcript is identified with one IPI ID, as we only investigated the probesets associated with protein-coding transcripts.

Statistics on the non-control, investigated and annotated probesets, together with the number of involved genes and proteins, are shown in Table 1. It is evident that the proportion of genes covered by our annotated probesets to those covered by all non-control probesets ('gene retaining percentage' in Table 1), 95.2% for MOE430A_2 and 85.4% for HG-U133A, are higher than the corresponding probeset retaining percentages, 83.5% and 68.8%, indicating that the gene coverage has only been slightly reduced by our filtering procedures. This observation is in support of our primary goal, that is to refine gene-level probeset annotations to transcript-level, but not to simply remove the poor-quality gene-level annotations.

Furthermore, we classified the probesets based on the way they linked to proteins. By checking the alignment results, the probesets in our annotation tables were divided into two groups, namely one-to-one and one-to-many. In our annotation table, a one-to-one probeset was linked to only a single protein, while a one-to-many probeset was linked to multiple proteins due to alternative splicing of one gene. The rest of the investigated probesets that were not linked to any protein were categorized into the third group of “one-to-null”. The statistics of these three groups are shown in Table 2. As we know, Affymetrix suffixes their probeset ID according to the probeset's specificity. The over ten types of probeset suffixes can be categorized into three groups: transcript-specific, with '_at', gene-specific, with '_a_at' or '_s_at', and other suboptimal probesets that may cross-hybridize with unrelated sequences. The first two groups are comparable to our one-to-one and one-to-many probesets respectively. However, we found that, out of a total of 21,097 and 16,213 investigated probesets in MOE430A_2 and HG-U133A respectively (Table 1), our one-to-one type accounts for only 53.7% and 43.3%, which were significantly lower than that of _at probesets, 74.0% and 72.6%, in the respective arrays. This suggests that some of _at probesets are not really specific for a transcript.

**Table 2:** One-to-one and one-to-many probesets in our annotation tables.

Investigated probesets		MOE430A_2	HG-U133A
Annotated probesets	One-to-one probesets	11327 (53.7% of investigated)	7014 (43.3% of investigated)
	One-to-many probesets	7567	8274
	
	*Sum*	18894	15288

One-to-null probesets		2203	925

**Total (Investigated probesets)**		**21097**	**16213**

To further clarify the issue of probeset specificity, we grouped the investigated probesets with the varied Affymetrix suffixes as well as our own categorizing system (Table 3). It is evident that overall one-to-one and one-to-many take up the majority of the _at group and the _a_at/_s_at group respectively, but there are some disagreements between the two classifications. For example, the Affymetrix _at group has 30.1% one-to-many probesets and 11.7% one-to-null probesets, suggesting that these so-called 'transcript-specific' probesets do not have the expected high specificity, and that they should be treated with caution in data analysis. On the other hand, the Affymetrix _a_at/_s_at group contains 42.0% one-to-one probesets. We attributed this mainly to the trimming of the originally false transcripts during the update of sequence information, as they may lead to the absence of the transcript targets of some probesets. Similarly, there are a large number of one-to-one probesets in the so-called Affy others group, 1,206 out of 2,619 for MOE430A_2 and 476 out of 1,555 for HG-U133A. These probesets, however, might not be really one-to-one mapping to the identified transcript targets, as our strategy was based on the premise that most probesets were specific for certain genes, and our blast was limited to the candidate transcripts associated with the probesets, but not the entire transcript pool.

**Table 3:** Comparison of the Affymetrix suffix categorization and our classification of probesets.

**Affy Suffix**	**Classification**	**MOE430A_2**	**HG-U133A**
_at	One-to-one	8473	4048
	One-to-many	4384	3035
	One-to-null	1697	625
	
	*Subtotal* *(% of Total)*	*14554* *(69.0%)*	*7708* *(47.5%)*

_a_at/_s_at	One-to-one	1648	2490
	One-to-many	2232	4363
	Not passing	44	97
	
	*Subtotal* *(% of Total)*	*3924* *(18.6%)*	*6950* *(42.9%)*

others	One-to-one	1206	476
	One-to-many	951	876
	Not passing	462	203
	
	*Subtotal* *(% of Total)*	*2619* *(12.4%)*	*1555* *(9.6%)*

**Total**		**21097**	**16213**

These subgroups of the investigated probesets with different Affymetrix suffixes indicate the imperfection of the Affymetrix suffix system, thus affirming the necessity of our transcript-level or protein-level probeset annotations. In fact, several other research groups have addressed the misleading nature of the Affymetrix suffix system and the imperfection of its standard annotation file, including some re-annotation works for array HG-U133 [14,18,19,23]. We will discuss these related works in detail in next section.

### Verification of the protein-level annotation and comparison with related annotations

Since the probesets were linked to transcripts and proteins through rigorous association and alignment procedures, the expression profiles of transcript-level synonymous probesets were supposed to be more consistent than those of gene-level synonymous probesets [14,15]. This was taken as the basis for the evaluation of our annotations.

**Figure 1:**
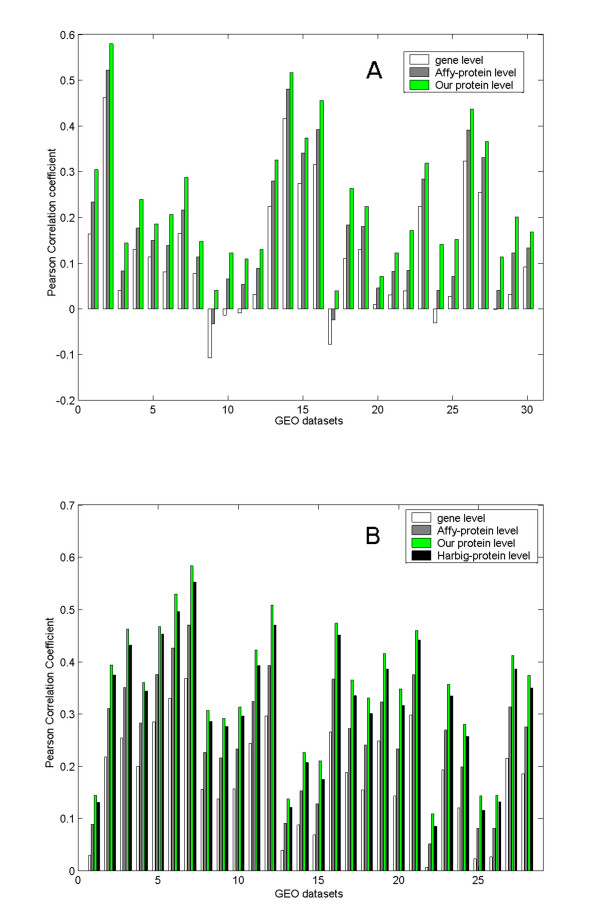
**Expression correlations of synonymous probesets**. X axis indicates expression dataset IDs, Y axis shows the Pearson Correlation Coefficient (PCC) of synonymous probesets summarized for each dataset. A) Expression correlations were calculated for the MOE430A_2 array based on 30 expression datasets. Synonymous probesets were compiled at three different levels: gene level, Affy-protein level, and our protein level. B) Expression correlations were calculated for the HG-U133A array based on 28 expression datasets. Synonymous probesets were compiled at four different levels: gene level, Affy-protein level, our protein level, and Harbig-protein level (according to Harbig et al.'s annotation work).

We downloaded 30 expression datasets assayed with MOE430A_2 from the Gene Expression Omnibus database (GEO) [24,25], and calculated the Pearson correlation coefficients (PCCs) of expression profiles of synonymous probesets at gene level and transcript level. Since transcript-level synonymous probesets in our work recognized a single protein-coding transcript variant identified with a unique IPI ID, transcript-level synonymous probesets were also named as protein-level synonymous ones. Practically, we grouped gene-level synonymous probesets according to gene ID, and protein-level ones according to IPI ID. The probeset-protein mapping tables provided at NetAffx, the official protein-level annotation of Affymetrix probesets [26], was used as comparison. Probesets corresponding to a single protein according to NetAffx were designated as 'Affy-protein' level synonymous probesets, which was a third level of synonymy. Within each synonymous group, all pair-wise PCCs were calculated and then summarized to one value indicating the expression correlation of this group. The expression correlations of all synonymous groups at one level were then averaged into an overall value for a dataset (see Method section for details). The correlations of synonymous groups for 30 datasets at three different levels were depicted in parallel in Figure 1A. It is noticeable that the synonymous probesets at Affy-protein level show higher correlations than those at gene level, but the synonymous ones based on our protein level annotation show even higher correlations consistently over 30 datasets (p < 0.05 for 30 datasets under student's t-test, see Additional file 2 for details). The comparison proves the rationality and necessity of our protein-level probeset annotation.

These results support the argument that it is more reliable to interpret microarray data at transcript level than at gene level. The inferiority of the Affy-protein level annotation to our annotation could be attributed to the technical details in their alignment and association procedures [26]. First, they used the representative mRNA sequence ('consensus' or 'exemplar' sequence of each probeset), instead of the probe sequences themselves, as the query sequence in the alignment. Second, they aligned against the GenBank non-redundant protein database, rather than a comprehensive pool of mRNA sequences. In microarray experiment hybridization takes place between the probe sequences immobilized on the array and the cDNA sequences from the sample, so one can deduce that the alignment between the representative mRNA sequences and the protein sequences cannot precisely simulate the hybridization between probes and mRNAs. Finally, NetAffx filtered the blast results according to a cutoff of E-value, which indicates the likelihood of the observed alignment by chance [27]. Although frequently adopted for sequence homology analysis in closely related species, E-value is not sensitive enough to grade the many well aligned targets from the same species. In our practice, we took the probe sequences as the query and the mRNA sequences as the alignment targets, and adopted the matching nucleotide proportion as the filtering criterion (see Methods).

A similar comparison was conducted for HG-U133A array, involving another transcript-level annotation by Harbig et al. [14]. Across the whole 28 datasets, the annotation by Harbig et al. showed advantage over Affy-protein-level annotation, while our transcript-level annotation performed best (p < 0.05 for 18 datasets under Student's t-test, see Figure 1B and Additional file 2). As our work was done two years later than Harbig et al.'s, the updated mRNA sequences in the probe-mRNA alignment is one of factors contributing to the increased performance. The other contributing factor is different approach we used to identify the mapping of probes to mRNA targets. Harbig et al. performed a two-phase blast: first, blast probeset target sequences against mRNA sequence pool, and then blast probe sequences against the retrieved mRNA sequences. Efficient as it was, this two-phase-blast strategy reduced the alignment precision as compared to our direct probe-against-mRNA blast strategy. Moreover, Harbig et al. accepted the mRNA with the highest average probe matches as the target of a probeset, even if the highest value could be suboptimal.

**Figure 2:**
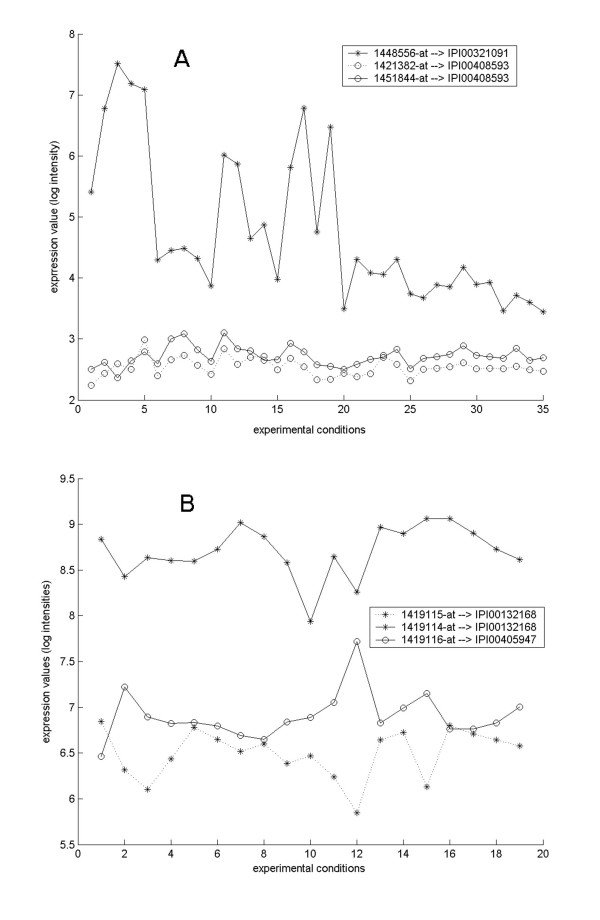
**Expression profiles of synonymous probesets**. A) Three probesets on the array MOE430A_2 are associated with the mouse gene Prlr. Probeset 1448556_at correlates to protein IPI00321091, or PRL-R3; probesets 1421382_at and 1451844_at both point to protein IPI00408593, or PRL-R2. GEO dataset GDS1277 was analyzed. B) Three probesets on the array MOE430A_2 are associated with the mouse gene Alg14. Probesets 149114_at and 149115_at both point to protein IPI00132168; probeset 1419116_at correlates to protein IPI00405947. GEO dataset GDS1076 was analyzed.

According to our data, the puzzling fact that probesets for one gene may show variable expression profiles can be clarified when viewing the data at the protein level. Such genes are likely to involve alternative transcript variants which are constitutively different in expression levels. We looked at two genes as examples. Shown in Figure 2A, three probesets in GDS1277 dataset, 1448556_at, 1421382_at and 1451844_at, were mapped to the mouse Prlr gene (GeneID: 19116), with the first probeset correlating to protein IPI00321091, or PRL-R3, and the other two correlating to protein IPI00408593, or PRL-R2. The Prlr gene was reported to encode at least seven isoforms of prolactin receptor precursor in mouse [16]. The two probesets corresponding to the same protein IPI00408593, showed similar expression profiles as expected, with a PCC value of 0.6738 (P = 4.57e-6); while the probeset corresponding to another isoform, IPI00321091, showed a significantly different expression profile, with PCCs of 0.2530 and -0.0331 separately for 1421382_at and 1451844_at (Figure 2A). These data were consistent with the previous report that these two isoforms were predominantly expressed in liver and kidney, where PRL-R3 was highly expressed and PRL-R2 was weakly expressed [16]. We noticed that the shape of the expression curve of PRL-R3 seemed to be somewhat comparable to those of PRL-R2, although PRL-R3 probeset presented an overall higher expression level. These phenomena may suggest that PRL-R3 and PRL-R2 are partially co-regulated so that they show a comparable expression pattern, but they are expressed at different levels probably due to diverse roles of the regulatory elements.

Shown in Figure 2B, three probesets in GDS1076 dataset, 1419114_at, 1419115_at and 1419116_at, were mapped to the mouse gene Alg14 (GeneID: 66789), with the former two corresponding to IPI00132168 and the latter one corresponding to IPI00405947. Both proteins were indicated in IPI as homologs of yeast asparagine-linked glycosylation 14 without any further information. We notice two interesting phenomena in this case. First, the two probesets correlating to a same protein do not show similar expression profiles. Instead, they behave like the probesets correlating to different variants characterized by a PCC value of 0.4080 (P = 0.0415). This issue might be due to some factors causing microarray hybridization efficiency shift. Secondly, according to our calculation, the expressions of these two variants are negatively correlated with PCC values of -0.7740 (P = 5.04e-5) for 1419116_at and 1419115_at, and -0.4402 (P = 0.0296) for 1419116_at and 1419114_at. So far there are no reports on expression regulation of these two transcript variants of gene Alg14, and no function reports of the corresponding protein isoforms. This negative correlation suggested that these two proteins might perform different roles thus should be distinctly annotated.

### Application of our new annotations the evaluation of the expression correlation between interacting proteins

In recent years, considerable efforts have been devoted to identifying and characterizing protein-protein interaction (PPI). Besides investigations on the molecular events involved in PPI, functional annotation of an unclassified protein according to its interacting partners is also an important topic [28]. Since it is too bold to infer protein functions according to the "majority rule" that utilizes only the PPI network structure [29,30], many studies integrate other data sources into the functional characterization of PPI, among which the gene expression data is the favorite [9,31,32]. All these works assumed that interacting protein pairs were characterized with higher expression correlation than random ones. However, previous investigations indicated that the relationship between expression correlation and PPI was weak on a genomic scale [33-35] although a recent work strengthened the association by integrating cross-species conservation information [10].

**Figure 3:**
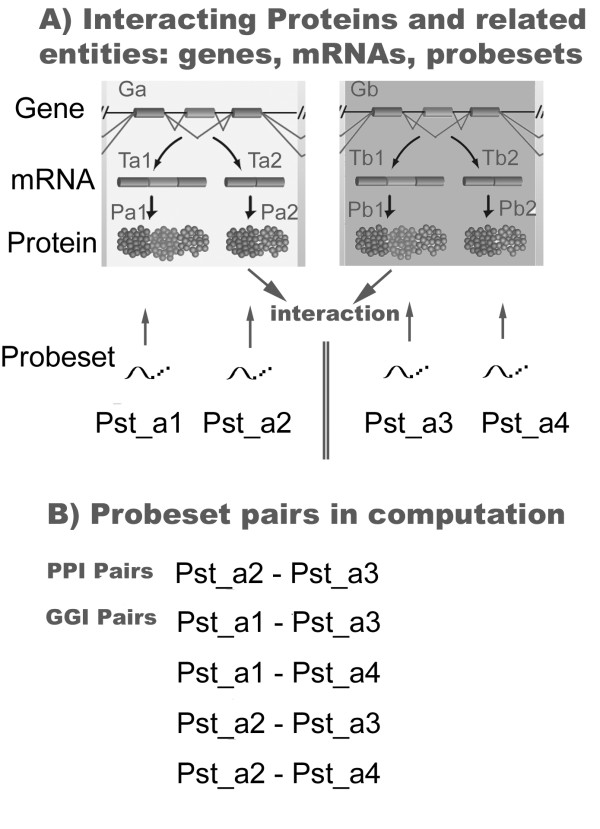
**Illustration of Entities and terminologies involved in protein-protein interaction**. A) Relationship among genes, transcripts, proteins and probesets. B) Two types of pairs mentioned in the text. PPI pair indicates the probeset pair correlating to a real interacting protein pair; while GGI pairs are those probeset pairs correlating to all possible cross-gene pairs. PPI may not exist in all cross-gene pairs.

We noticed that in these genome-scale studies PPI information was always first converted to gene pairs, after which the Pearson correlations of the probeset pairs corresponding to the gene pairs were evaluated. That is, the analysis targets were expanded from real interacting protein pairs to all possible cross-gene protein pairs for which interaction may not always exist. As illustrated in Figure 3, suppose we have gene a (abbreviated to Ga) and gene b (Gb), with Ga encoding protein a1 (Pa1) and protein a2 (Pa2), Gb encoding protein b1 (Pb1) and protein b2 (Pb2). Among these protein variants, only proteins Pa2 and Pb1 interact with each other, while the other three possible cross-gene interactions, including Pa1-Pb1, Pa1-Pb2 and Pa2-Pb2, do not really happen. The four probesets, Pst_a1, Pst_a2, Pst_b1, and Pst_b2, recognize transcript variants Ta1, Ta2, Tb1 and Tb2 respectively, producing proteins Pa1, Pa2, Pb1 and Pb2. In the conventional genome-scale studies mentioned above, besides the probeset pair (Pst_a2, Pst_b1) corresponding to the real interacting protein pair, the other three cross-gene pairs, (Pst_a1, Pst_b1), (Pst_a1, Pst_b2) and (Pst_a2, Pst_b2), were also included, which would blunt the expression correlation between the real interacting entities according to our preceding observations. We propose that this might partly explain the weak coherency between PPI and expression correlation.

In order to validate our proposition, we investigated the relationship between protein interactions and expression correlation at both gene-level and protein-level perspectives, using 1,037 interacting protein pairs from the HPRD [36] and 28 microarray datasets assayed with HG-U133A from the GEO. As depicted in Figure 3, the probeset pairs corresponding to all possible cross-gene protein pairs are termed as GGI pairs, out of which only the probeset pair corresponding to the real PPI, such as Pst_a2-Pst_b1, is a PPI pair. We calculated PCCs of both PPI pairs and GGI pairs for all available 1,037 interactions, evaluated the statistical significances of these PCC values under one-tailed t-test, and estimated the corresponding false discovery rates (FDR) using the SPLOSH FDR estimation method [37].

**Figure 4:**
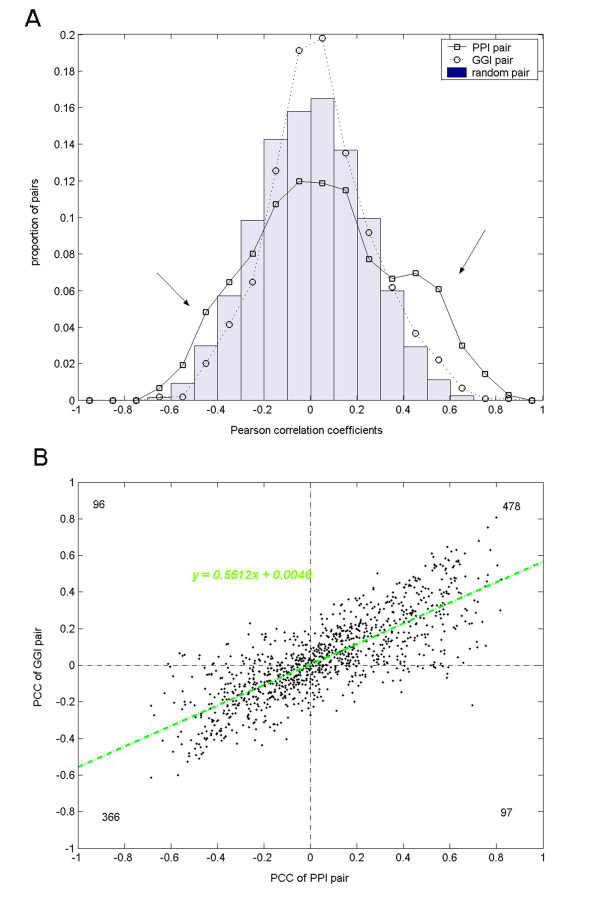
**Pearson correlation coefficients of PPI pairs and corresponding Gene-Gene Interaction GGI pairs**. 1,037 interacting protein pairs from the HPRD were analyzed based on the GEO dataset GDS987. A) Distributions of PCC values for PPI pairs, GGI pairs and random pairs. The two arrows indicate the regions where expression correlations were strengthened by the PPI PCC calculation. (B) Scatter plot of PCC values of PPI pairs vs. those of corresponding GGI pairs. X-axis indicates the PCC values of PPI pairs, and y-axis indicates the PCC values of the corresponding GGI pairs. A linear regression resulted in a regression line and a regression formula, which were shown in green. Totals within each quadrants were marked with black numbers at the four corners.

For all datasets, we observed strengthened expression correlations between interacting proteins under the PPI schema relative to the GGI schema. Taking the GDS987 dataset including 41 arrays as an example (Figure 4A and Additional file 3), while it is difficult to tell the distribution of GGI PCC values from that of the random ones, the PPI PCC values exhibit a distinguishable distribution biased to larger absolute correlation values. We compared the distributions for positive correlations and negative correlations separately. Out of the 1037 interactions, 575 PPI PCC values were positive with a mean value of 0.2924, which was significantly larger than the mean of the 574 positive GGI PCC values, 0.2387 (Kolmogorov-Smirnov test p value is 8.2E-8); On the other hand, the mean values of the other 462 negative PPI PCC values and the 463 negative GGI PCC values were -0.2263 and -0.153, respectively, and the former was significantly smaller than the latter (Kolmogorov-Smirnov test p value is 9.9E-12). Summarizing the comparisons at the positive side and the negative side, we conclude that the expression correlation between interacting proteins is strengthened when the non-interacting protein pairs are excluded from the interacting gene pairs, that is, with the PPI PCC calculation in place of the conventional GGI PCC calculation.

In Figure 4A, we notice that the negative correlation is also strengthened by the PPI PCC calculation as well as the positive correlation. That is to say, PPI pairs seem to be either positively correlated or negatively correlated, but not exclusively 'co-expressed' as previous publications reported [10]. This phenomenon is more evident when we examine the PCC values for each coupled PPI pair and GGI pair. Figure 4B shows a scatter plot of the PPI PCC values versus the corresponding GGI PCC values. It is evident that most points fall into the 1st and the 3rd quadrants, indicating that each pair of PPI PCC value and GGI PCC value tends to have the same signs. The scatter plot suggests a linear relationship between the PPI PCCs and GGI PCCs, and indeed we get a linear regression formula, y = 0.5612x + 0.0046, at high confidence (p < 2e-16). Since the estimated coefficient, 0.5612, is far less than 1, we may conclude that the absolute PCC values of PPI pairs are often larger than those of the corresponding GGI pairs. So the PPI PCC calculation preserves the original positive or negative correlation tendency revealed by the conventional GGI PCC calculation, and strengthens it with larger absolute correlation values. Such correlation tendencies between interacting proteins, especially those negative ones, would very likely be submerged under the background correlations of random pairs if the non-interacting protein pairs are included in the analysis.

Similar observations were made over all datasets (see Additional file 4 and Additional file 5). Given the results from all 28 datasets, we were also able to compare the PPI PCC calculation and GGI PCC calculation at a higher level. Under a FDR threshold of 0.1, the list of significant PPI pairs or GGI pairs from each dataset was determined. Based on these lists, we counted the significantly correlated PPIs or GGIs (termed 'significant PPIs' or 'significant GGIs') over each dataset, and the datasets on which a PPI or GGI demonstrates significant correlation (termed 'PPI-significant datasets' or 'GGI-significant datasets'). The former is a 'twenty-eight by two' table, shown in Table 4; the latter is a '1,037 by two' table, shown fully in Additional file 4 and partly in Table 5. In both summary tables, we find that the statistics of PPI PCC calculation are mostly larger than the counterparts of GGI PCC calculation, indicating that PPI PCC calculation can detect more correlated PPI pairs in a certain experiment setting (Table 4), and that it can detect the correlation tendency across more experiment settings (Table 5).

**Table 4:** Number of significantly correlated PPIs or GGIs found from each dataset.

**GEO** **Data Set**	**Significant** **PPIs**	**Significant** **GGIs**
GDS1023	42	2
GDS1036	70	13
GDS1062	148	13
GDS1064	46	20
GDS1067	167	86
GDS1096	248	28
GDS1209	344	68
GDS1220	297	36
GDS1257	54	26
GDS1259	20	4
GDS1282	84	24
GDS1321	121	18
GDS1331	18	2
GDS266	7	0
GDS268	3	0
GDS395	476	35
GDS558	160	26
GDS564	13	0
GDS596	737	455
GDS690	462	163
GDS715	866	754
GDS737	7	1
GDS760	109	19
GDS810	33	4
GDS914	13	1
GDS946	22	5
GDS987	464	115
GDS999	263	56
GDS999	263	56

Protein pairs with the false discovery rate of PCC value less than 0.1 were deemed as significantly correlated pairs. 'Significant PPIs' are pairs detected by the PPI calculation; 'Significant GGIs' are those detected by the GGI calculation.

**Table 5:** Number of datasets on which a PPI or GGI demonstrate significant correlation.

IPI1	IPI2	Gene1	Gene2	PPI-significant datasets	GGI-significant datasets
IPI00003326	IPI00015161	ARL2	PDE6D	15	7
IPI00003894	IPI00019930	RNF11	UBE2D1	17	1
IPI00007411	IPI00021831	AKAP11	PRKAR1A	15	2
IPI00008529	IPI00008527	RPLP2	RPLP1	25	5
IPI00011118	IPI00026689	RRM2	CDC2	17	16
IPI00013871	IPI00011118	RRM1	RRM2	14	10
IPI00015952	IPI00029012	EIF4G2	EIF3S10	14	3
IPI00016910	IPI00290460	EIF3S8	EIF3S4	16	10
IPI00018350	IPI00184330	MCM5	MCM2	16	15
IPI00021700	IPI00026689	PCNA	CDC2	14	13
IPI00022865	IPI00026689	CCNA2	CDC2	16	15
IPI00026689	IPI00015105	CDC2	CKS2	15	13
IPI00027462	IPI00007047	S100A9	S100A8	23	17
IPI00028266	IPI00026689	CCNB2	CDC2	17	15
IPI00165506	IPI00025616	POLDIP2	POLD2	14	2
IPI00246058	IPI00025277	PDCD6IP	PDCD6	16	1
IPI00290461	IPI00029012	EIF3S1	EIF3S10	15	4
IPI00291006	IPI00018206	MDH2	GOT2	16	11
IPI00294696	IPI00026689	CCNB1	CDC2	17	14
IPI00306708	IPI00026689	PBK	CDC2	16	14
IPI00328118	IPI00026689	SPAG5	CDC2	15	10
IPI00604664	IPI00291006	NDUFS1	MDH2	18	10
IPI00647217	IPI00552920	SKIV2L2	EXOSC8	18	2

A PPI-significant dataset is a dataset where the false discovery rate of the PCC value for the PPI pair is less than 0.1, while a GGI-significant dataset is a dataset where the false discovery rate of the PCC value for the GGI pair is less than 0.1. Only PPI pairs with more than 14 'PPI-significant datasets' datasets are shown. The full table is provided in Additional file 4.

The same experiments were also implemented with 274 PPI pairs extracted from the IntAct database [38], and similar conclusions were obtained. More details can be found in Additional file 6 and Additional file 7.

## Discussion

In this work, we re-annotated the probesets of two widely used Affymetrix arrays, MOE430A_2 and HG-U133A, *via *proper association and rigorous alignment procedures in a transcript perspective, and demonstrated the necessity and advantage of exploring microarray data at the transcript or protein level, instead of the conventional gene level.

Although Affymetrix utilized the most complete information available at the time of array design, tremendous progress in genome sequencing and annotation in recent years renders existing probeset designs and target identifications suboptimal. In recent years, there have been continuous reports on systematic false expression signals of Affymetrix probesets [39], spurious expression correlation caused by cross hybridization [18], and expressional inconsistency among different microarray platforms or even different generations of one platform [40-43]. A few research groups performed probe-against-mRNA blast similar to ours [22,42,44], but mostly they centered around UniGene [45] and therefore improved the accuracy of annotation only at gene level. A major trend among these efforts was to redefine probesets so that probes matching the same molecular target were placed into custom probesets, as proposed by [19,23,39,42], but as the authors of [19] pointed out, 'these transcript-targeted probesets are not transcript-specific, as probesets targeting transcripts from the same gene may share many or even all probes'. Thus the probe re-organization strategy may be used to make distinction at the level of genes, but not at the level of transcripts or splice variants [18]. Besides, this strategy takes the probe-level intensity file (the CEL file) as a prerequisite, however only around half of the expression datasets deposited in public databases like GEO were found with CEL files.

In order to make distinction precisely at the transcript level, we preserved the classical Affymetrix probesets, but distinguished them among their alternatively spliced transcript targets according to the consistent alignments of probes against up-to-date mRNA sequences. Our annotation table clearly divides the Affymetrix probesets into three groups with increased transcript-level specificity (reliability): one-to-null probesets that do not recognize any transcript, one-to-many probesets that hybridize to multiple alternative transcript variants of the intended gene, and one-to-one probesets that hybridize to unique alternative transcript variants of the intended gene. We discriminate the intended alternative transcript variants of Affymetrix probesets based on the NetAffx's gene-level annotation for the first time. Given the fact that existing solutions are accompanied with imperfections and no consensus has been reached on an overwhelming strategy, our alternative solution to the problematic standard annotation points out a new way to improve the interpretation and exploitation of Affymetrix microarray data.

Although the transcript collections were not identical and the reannotation strategies differed more or less, we made out some similar discoveries to previous reports. For example, Harbig *et al*. found that a number of probesets did not detect any transcript and attributed this phenomenon to the elimination of the target sequence in the process of sequence update [14]. In our study, altogether 16.5% and 31.2% of non-control probesets in the MOE430A_2 and HG-U133A arrays were not found with any transcript targets in the pool of GenBank, RefSeq and Ensembl. Using newer and larger collection of transcript sequences, we even obtained a quite similar statistics of the percentage of 'multiple-targeting' probesets to that estimated in a foregoing study [18], specifically 54.6% for MOE430A_2 and 54.1% HG-U133A (see Table 2). The significant mutual agreement among the related researches justifies the necessity to set up an improved annotation mechanism of the Affymetrix probes in the face of the continual growth of genomic and transcriptomic knowledge, ideally at transcript or protein level.

Over the past few years, the analysis of alternative splicing has emerged as an important new field in bioinformatics, and several recent large-scale studies have shown that alternative splicing can be analyzed in a high-throughput manner using DNA-microarray methods [46,47]. Most of these studies used arrays particularly manufactured for analyzing alternative splicing, such as genomic tiling array and exon-exon junction array. Constructed without any *priori *knowledge of the possible exon content of a genomic sequence, the genomic tiling array [48,49] is in principle capable of detecting novel alternative splicing events of diverse types, but it is in doubt whether their data will be readily interpretable as successful experiences remain insufficient [46]. On the other hand, although designed particularly to address the alternative splicing issue, exon and exon-exon junction arrays [49] were reported to be plagued by problematic probe specificity and unsatisfying hybridization efficiency because of the necessity of probe coverage across the full length of the gene (including 5' end) [5]. Many questions about the reproducibility of the amplification protocol, the quantitative accuracy, and the data analysis need to be addressed as a prerequisite to reliable quantitative analysis using these splicing arrays [50]. Given the current imperfection of splicing array techniques and inconvenience in deciphering their generated data, it is an economic way to do large-scale investigations of alternative transcribing events with standard gene expression arrays, provided that the recognizing targets of the probes can be rigorously defined at the transcript level. Hu *et al*. proposed a primitive analysis method to explore alternative splicing with Affymetrix 3' gene expression arrays, though they regretted that only alternative splicing biased toward the 3'end of the gene can be detected in their way [51]. In the present paper, we conducted a large-scale alignment of the probe sequences in traditional gene expression arrays against the currently most comprehensive collection of transcript sequences, highlighting the probesets mapping to unique alternative transcripts unambiguously. For each of the two Affymetrix expression arrays tested in this study, we found over 40% of all probesets could be mapped to transcripts in a one-to-one manner, so our work strongly validate that it is feasible to analyze alternative splicing using traditional gene expression arrays. While the foregoing work contributed by Hu *et al*. remains as a qualitative analysis method aiming at detecting novel alternative splicing events, our work gives explicitly the relationship between the probesets and the currently known alternative transcript variants, which can be immediately exploited to facilitate quantitative analysis of alternative variants. As our mapping relationships are defined for the standard probesets of the traditional gene expression arrays, they can be conveniently exploited as the standard NetAffx annotation information, without any *ad hoc *influence on the widely applied experiment protocols or the routine data processing algorithms. In the demonstrative implementations of the novel annotation tables, we actually observed several examples of negatively correlated alternative variants (see Figure 2B for one of them), which will shed light on further studies of expression regulation of alternative transcript variants.

## Conclusion

To sum up, we re-annotated two popular Affymetrix gene expression arrays, MOE430_2 and HG-U133A, in a transcript-level perspective, aiming at identifying probesets' detecting targets precisely at the transcript level. Although previous works addressed similar issues [14,15,18,19,22,23], we are the first to rigorously link existing Affymetrix probesets to their specific transcript targets and their corresponding proteins. Armed with this new annotation, we re-examined a number of previous studies, 30 datasets for MOE430_2 and 28 datasets for HG-U133A from GEO, and revealed increased expression consistency among synonymous probesets and closer expression correlation among interacting proteins. This transcript-level annotation of Affymetrix probesets allows for a more reliable gene expression data analysis and a more accessible protein-level correlation study.

## Methods

### Sequences and related information of Affymetrix probesets

The Affymetrix 3' eukaryotic gene expression analysis arrays MOE430A_2 and HG-U133A were selected for this study. Probe sequence files and corresponding annotation files, 'Mouse430A_2_annot_csv.zip' (annotated on 2005-12-19) and 'HG-U133A_annot_csv.zip' (annotated on 2006-04-11), were downloaded from Affymetrix website [52]. Also downloaded there were the NetAffx probeset-protein mapping files for MOE430A_2 (file 'Mouse430A_2_blast_csv', updated on 2005-12-18) and HG-U133A ('HG-U133A.na21.blast.csv.zip', updated 2006-04-11), which were the blast results of the representative mRNA sequence of probes against protein sequence databases [26].

### Sources of mRNA transcripts

GenBank: mRNA sequences from CoreNucleotide division of NCBI Nucleotide database were obtained via the Entrez Nucleotide search [53] on April 10^th^, 2006. For mouse, this dataset comprises 1,582,211 sequences, with 1,521,234 from DDBJ, 6,506 from EMBL and 54,471 from GenBank. For human, there are totally 201,206 sequences, with 41,128 from DDBJ, 62,701 from EMBL and 97,377 from GenBank. File 'gene2accession' (updated on 2006-03-28), downloaded from Entrez Gene [54], provides the mapping relationship between the CoreNucleotide sequences, Entrez Gene IDs, and protein sequence accessions.

RefSeq: 55,832 mouse mRNA sequences and 40,530 human mRNA sequences were obtained from the RefSeq database [55]. Mapping relationships between RefSeq mRNA accessions, RefSeq protein accessions, and Entrez GeneIDs were extracted from related flat files 'mouse.rna.gbff.gz' and 'human.rna.gbff.gz', which were downloaded from RefSeq in April 2006.

Ensembl transcripts: 37,854 mouse transcript sequences were obtained from the Ensembl database (release 38) [56]. Mapping tables between Ensembl Gene ID, Ensembl Transcript ID, and Ensembl Peptide ID were obtained from Ensembl martview [57].

### IPI entries and their mappings to external sequence accession numbers

IPI entries and their mappings to external protein accession numbers were acquired from the International Protein Index (IPI) database [21] (release 3.17). Also obtained there were the mapping relations between IPI numbers and transcript IDs (GenBank, RefSeq, and Ensembl). The counterpart file for human was downloaded there too (release 3.16).

### Expression datasets from Gene Expression Omibus

Microarray datasets were downloaded from the Gene Expression Ominibus on April 15, 2006. Array MOE430A_2, indexed as GPL339, was associated with 2,276 samples in GEO, ranking the second among all registered Affymetrix mouse arrays. All 31 GDS datasets profiled with MOE430A_2 were used in the analyses except for GDS1057, which contains only two samples. Array HG-U133A, indexed as GPL96, was associated with 8,698 samples in GEO, ranking the first among all registered Affymetrix human arrays. For our analysis, we downloaded 31 GDS datasets with largest sample sizes, and used 28 of them in our analyses, excluding GDS534, GDS1329, and GDS1324 as they are in a data format inconsistent with the others. Details about the used datasets can be found in our Additional file 8.

### PPI datasets from IntAct and HPRD

Two well-known databases, IntAct [58] and HPRD [59], provide the PPI information for this study. We downloaded 68,035 human PPIs from HPRD (updated 2006-06-01) and 12,301 from IntAct (updated 2006-05-12), respectively.

### An alternative annotation of the Affymetrix U133 Plus 2.0 array

A recently proposed transcript-level annotation of the Affymetrix U133 plus 2.0 array was obtained from the H. Lee Moffitt cancer center and research institute [60], which was used for comparison with our transcript-level annotation of HG-U133A array.

### Generation of a new transcript-level annotation table for Affymetrix array

Out of the total 22,690 and 22,283 probesets in arrays MOE430A_2 and HG-U133A, respectively, 64 and 68 control probesets were firstly removed. The remaining probesets were associated with genes according to the probeset-gene mapping information provided in Affymetrix's standard annotation file. The probeset-transcript mapping relationships were obtained based on the gene-mRNA mapping tables from GeneBank, RefSeq and Ensembl. In the process, we only included probesets that were identified with one Entrez Gene ID or one Ensembl gene ID. We ignored the probesets that were associated with multiple entities or no entity in the two gene-centric databases, since their gene-level specificity appears doubtful in the standard annotation file. This filtered out 3.2% and 5.2% of non-control probesets in MOE430A_2 and HG-U133A, respectively. For the rest of the probesets, we linked the candidate transcript targets to their corresponding protein entries in IPI database. IPI is currently the least redundant yet most complete protein database for featured species, with one protein sequence matching each transcript variant. Those probesets of which transcript targets do not have any protein counterparts were also excluded from the following blast validation in order to focus our attention to the transcripts with well-characterized functions at protein level. The remaining probesets, 21,097 for MOE430A_2 and 16,213 for HG-U133A, were selected for the BLAST procedure.

We then filtered the candidate probeset-mRNA mapping relationships by aligning probe sequences in these probesets against their corresponding transcripts. Probes were blasted against their candidate mRNA targets using the bl2seq program [61], and the probe to transcript matches were accepted if no more than one mismatch was found. At the level of probesets, the probeset to transcript matches were accepted only if more than 90% of all probes within a probeset (over 10 probes for the typical 11-probeset) were mapped to the transcript in the same orientation.

The probeset-transcript-protein links related to the above probesets passing BLAST filter were retrieved. After reducing the redundancy information of multiple transcripts corresponding to the same IPI, we finally obtained rigorous probeset annotation files for Affymetrix arrays MOE430A_2 and HG-U133A. There are two types of probesets in the new annotation file: one-to-one probesets, where one probeset maps to only one IPI ID; and one-to-many probesets, where one probeset maps to two or more IPI IDs. Only the one-to-one probesets were used in the subsequent analyses.

### Evaluating expression consistencies within synonymous probesets

We grouped gene-level synonymous probesets according to gene ID (gene-level), and protein-level ones according to IPI ID (protein-level). Additionally, probesets corresponding to a single protein according to NetAffx probeset-protein mapping tables (see Materials) are grouped as 'Affy-protein' level synonymous probesets. In the case of the HG-U133A array, we included a fourth level, the 'Harbig-protein level', for comparison. The Harbig-protein level reflects the probeset-protein association transformed from a recent alternative annotation of the Affymetrix U133 plus 2.0 array [14], also proposed in a transcript-level perspective. The downloaded annotation file mapped 33,579 probesets to 287,791 GenBank mRNA accessions, among which 21,669 were found on HG-U133A array, mapped to 186,085 GenBank mRNA accessions. The probeset-mRNA associations related to HG-U133A array were further linked to IPI IDs, finally giving rise to 26,960 probeset-IPI mapping relationships among which 12,146 were one-to-one. The following treatments were the same as those implemented to the standard gene-level annotation, the NetAffx Protein annotations, and our novel annotations.

30 and 28 expression datasets were selected from GEO respectively for MOE430A_2 and HG-U133A, and the original intensity data within each GEO dataset (GDS) were transformed to log 2 base and normalized to a constant median across all samples. For a synonymous group, if the expression values of all probesets in all samples were no larger than the constant median value, the probesets in this group were regarded not moderately expressed, and their expression profiles not informative enough. Therefore, we only kept the synonymous groups with at least one expression value above the constant median value, similar to the filtering procedure used by Tian *et al*. [62]. For the remaining synonymous groups, Pearson correlation coefficients (PCCs) were calculated for the expression profiles of each probeset pair. The minimum value of these PCCs was taken as a measurement of the expression coherence of this group. We used the minimum aggregation because the gene level synonymous probesets gave rise to within-protein PCCs (which are theoretically higher) and across-protein PCCs (which are theoretically lower), and the former was identical to the result of the protein-level synonymous group. In such a setting, the maximum did not result in any difference, and the average aggregation was not as sensitive as the minimum in terms of differentiating the two groups, so we adopted the minimum aggregation.

Finally, the mean of the expression coherences of all synonymous groups over a dataset was calculated. In this way we obtained an evaluation of expression consistencies within synonymous probesets for a microarray dataset, and may compare the expression consistencies at the three levels over different microarray datasets.

### Investigating expression correlations between interacting protein pairs

Given protein-protein interaction data from HPRD or IntAct, we first transformed the binary relations of protein accessions to IPI-IPI pairs, and also got the corresponding Gene-Gene pairs. For each PPI, we assembled the PPI probeset pairs and the GGI probeset pairs as illustrated in Figure 3, where PPI pairs are those associated with the interacting IPI IDs while GGI pairs are those associated with the corresponding Gene IDs. For all probeset pairs associated with the IPI-IPI pair (PPI pairs) and those associated with the corresponding gene-gene pair (GGI pairs), the PCCs were calculated and averaged into a PPI PCC and GGI PCC, respectively. These PCCs of interacting pairs were further calculated to obtain the accompanying false discovery rates using the SPLOSH FDR estimation method.

The distributions of the PPI PCCs and the GGI PCCs were plotted in a same figure to show the contrast (Figure 4A). In addition, a background distribution of the PCCs of random probeset pairs was overlaid on the same figure. We let the number of random pairs equal to the number of PPI or GGI pairs, but repeated the process of calculating random PCC distribution 20 times and averaged over the 20 separate random distributions in order to cut down on random fluctuation. Within each run of calculating random PCC distribution, we randomly compiled 2 × *n *(*n *= 1037 or 274, for HPRD or IntAct, respectively) pairs of probesets, where each two probeset pairs formed a group. The two PCCs of each group were firstly averaged into a group-level PCC, and the distribution was calculated over the *n *group-level PCCs. The group-level averaging was devised to mimic the counterpart operation in PPI PCC or GGI PCC calculation.

## Abbreviations

BLAST: The Basic Local Alignment Search Tool

GEO: Gene Expression Omnibus

GGI: gene-gene interaction

HG-U133A: Human Genome U133A Array

HPRD: Human Protein Reference Database

IPI: International Protein Index

MOE430A_2: Mouse Genome 430A 2.0 Array

PCC: Pearson correlation coefficient

PPI: Protein-protein interaction

## Authors' contributions

YYL, KT, and YXL conceived and designed the experiments. HY, FW, KT performed the experiments. HY, YYL, FW, and LX analyzed data. YYL, HY, and LX wrote the manuscript. All authors read and approved the final manuscript.

## Supplementary Material

Additional file 1 Novel annotation tables. Novel annotation files at transcript level, for MOE430A_2 and HG-U133A arrays. Click here for file

Additional file 2 Expression correlations of synonymous probesets. Expression consistency within synonymous probeset groups for 30 (assayed with MOE430A_2) and 28 (assayed with HG-U133A array) GEO datasets, with synonymity defined at different levels: gene level, Affy-protein level, and our protein level. For HG-U133A array, a recent related work (Harbig et al. 2005 NAR) was also adopted for comparison. Click here for file

Additional file 3 Pearson correlation coefficients (PCCs) calculated on dataset GDS987. Pearson correlation coefficients (PCCs) of PPI pairs and corresponding GGI pairs. Calculated for 1037 HPRD protein-protein interactions using the GEO dataset GDS987. Click here for file

Additional file 4 Expression correlation of PPI and GGI pairs from HPRD. PPI and GGI Pearson correlation coefficients for 1037 HPRD PPIs. Click here for file

Additional file 5 Summary of expression correlations of 1037 HPRD PPI and GGI pairs. 1) Distribution plots for PPI and GGI Pearson correlation coefficients (PCCs), one for a GDS dataset. PCCs of random pairs form the background distribution. 2) Scatter plots of PPI and GGI PCCs. Click here for file

Additional file 6 Expression correlation of PPI and GGI pairs from IntAct. PPI and GGI Pearson correlation coefficients for 274 IntAct PPIs. Click here for file

Additional file 7 Summary of expression correlations of 274 IntAct PPI and GGI pairs. 1) Distribution plots for PPI and GGI Pearson correlation coefficients (PCCs), one for a GDS dataset. PCCs of random pairs form the background distribution. 2) Scatter plots of PPI and GGI PCCs. Click here for file

Additional file 8 Information on used GDS datasets. Accession numbers of GEO datasets used in the analysis. Click here for file
